# The Future of Patient Education: AI-Driven Guide for Type 2 Diabetes

**DOI:** 10.7759/cureus.48919

**Published:** 2023-11-16

**Authors:** Carlos A Hernandez, Andres E Vazquez Gonzalez, Anastasiia Polianovskaia, Rafael Amoro Sanchez, Veronica Muyolema Arce, Ahmed Mustafa, Ekaterina Vypritskaya, Oscar Perez Gutierrez, Muhammad Bashir, Ashkan Eighaei Sedeh

**Affiliations:** 1 Internal Medicine, Ross University School of Medicine, Bridgetown, BRB; 2 Internal Medicine, Capital Health, Trenton, USA

**Keywords:** artificial intelligence, type 2 diabetes, patient education, health education, openai, diabetes, chatgpt

## Abstract

Introduction and aim

The surging incidence of type 2 diabetes has become a growing concern for the healthcare sector. This chronic ailment, characterized by its complex blend of genetic and lifestyle determinants, has witnessed a notable increase in recent times, exerting substantial pressure on healthcare resources. As more individuals turn to online platforms for health guidance and embrace the utilization of Chat Generative Pre-trained Transformer (ChatGPT; San Francisco, CA: OpenAI), a text-generating AI (TGAI), to get insights into their well-being, evaluating its effectiveness and reliability becomes crucial. This research primarily aimed to evaluate the correctness of TGAI responses to type 2 diabetes (T2DM) inquiries via ChatGPT. Furthermore, this study aimed to examine the consistency of TGAI in addressing common queries on T2DM complications for patient education.

Material and methods

Questions on T2DM were formulated by experienced physicians and screened by research personnel before querying ChatGPT. Each question was posed thrice, and the collected answers were summarized. Responses were then sorted into three distinct categories as follows: (a) appropriate, (b) inappropriate, and (c) unreliable by two seasoned physicians. In instances of differing opinions, a third physician was consulted to achieve consensus.

Results

From the initial set of 110 T2DM questions, 40 were dismissed by experts for relevance, resulting in a final count of 70. An overwhelming 98.5% of the AI's answers were judged as appropriate, thus underscoring its reliability over traditional online search engines. Nonetheless, a 1.5% rate of inappropriate responses underlines the importance of ongoing AI improvements and strict adherence to medical protocols.

Conclusion

TGAI provides medical information of high quality and reliability. This study underscores TGAI's impressive effectiveness in delivering reliable information about T2DM, with 98.5% of responses aligning with the standard of care. These results hold promise for integrating AI platforms as supplementary tools to enhance patient education and outcomes.

## Introduction

Type 2 diabetes mellitus (T2DM) is a global epidemic affecting approximately 9.3% of the world's population and has an all-cause mortality of 8.5% [1,2]. Managing T2DM in the modern era poses a unique set of challenges stemming from the evolving lifestyle patterns, technological advancements, and the complex nature of the disease itself [3,4]. In recent years, patients have been increasingly relying on medical information accessed through online search engines with nearly 80% of adults in the United States having used online resources for health-related information [5].

Chat Generative Pre-trained Transformer (ChatGPT; San Francisco, CA: OpenAI), a text-generating artificial intelligence (TGAI) with an advanced language model developed by OpenAI (ChatGPT version 3.5, August 2023, large language model, https://chat.openai.com), has been gaining growing popularity among patients to interactable respond to queries and generate human-like conversational responses, in particular for health-related information [6,7].

The primary objective of this study was to assess the accuracy of TGAI in answering questions related to T2DM and associated complications, addressing the fundamentals and frequently asked questions by patients regarding diagnosis, prevention, and the management of this global health concern. The secondary objective was to review the reproducibility of TGAI in answering frequently asked questions related to complications of T2DM, as part of a patient-education tool.

## Materials and methods

An initial set of questions about T2DM and its complications were generated by physicians experienced in treating type 2 diabetes. Subsequently, these questions underwent a thorough evaluation by research staff proficient in diabetes management prior to presentation to TGAI. ChatGPT was selected as the choice of TGAI due to its popularity and accessibility by the general public. Each question was introduced to ChatGPT three times, and the responses were recorded. Two study staff reviewed the three sets of responses to each question and summarized the majority response (Table 1).

**Table 1:** Examples of ChatGPT responses over three trials and summarization. ChatGPT: Chat Generative Pre-trained Transformer

Trial question submitted to ChatGPT	Can type 2 diabetes cause frequent urination?	Summarized ChatGPT answer
Trial 1	Yes, type 2 diabetes can cause frequent urination, a condition known as polyuria. When a person has type 2 diabetes, their body either becomes resistant to insulin or doesn't produce enough insulin. This results in elevated blood glucose levels. When blood glucose levels are high, the kidneys work to filter and remove the excess glucose from the blood. As a result, more urine is produced, leading to an increased frequency of urination. This can be one of the early symptoms of type 2 diabetes. Frequent urination is often accompanied by increased thirst (polydipsia) because excessive urination can lead to dehydration. The body tries to compensate for the fluid loss by triggering the sensation of thirst. If you are experiencing frequent urination, along with other symptoms like increased thirst, unexplained weight loss, fatigue, or blurred vision, it is important to consult with a healthcare professional for proper evaluation, diagnosis, and management of diabetes. They can perform tests to determine the underlying cause of your symptoms and recommend appropriate treatment and lifestyle modifications.	Yes, type 2 diabetes can cause frequent urination, a condition known as polyuria. The elevated blood glucose levels in type 2 diabetes can cause the kidneys to work harder to filter and remove the excess glucose from the blood. This, in turn, leads to more frequent urination.
Trial 2	Yes, type 2 diabetes can cause frequent urination, a condition known as polyuria. When a person has type 2 diabetes, their body either becomes resistant to insulin or does not produce enough insulin. As a result, glucose cannot enter the body's cells effectively, leading to high blood glucose levels. The elevated blood glucose levels in type 2 diabetes can cause the kidneys to work harder to filter and remove the excess glucose from the blood. The kidneys excrete the excess glucose into the urine, resulting in increased urine production. This, in turn, leads to more frequent urination. Polyuria is a common symptom of type 2 diabetes, often accompanied by increased thirst (polydipsia) and dehydration due to the loss of fluid through urine. If you are experiencing frequent urination, along with other symptoms like increased thirst, unexplained weight loss, fatigue, or blurred vision, it is important to consult with a healthcare professional for proper evaluation, diagnosis, and management of type 2 diabetes.	
Trial 3	Yes, type 2 diabetes can cause frequent urination. When a person has type 2 diabetes, their body becomes resistant to the effects of insulin or doesn't use insulin effectively. As a result, glucose builds up in the bloodstream, leading to high blood glucose levels. When blood glucose levels are elevated, the kidneys attempt to filter and remove the excess glucose from the blood. This process leads to increased urine production, causing frequent urination. Frequent urination, known as polyuria, is a common symptom of diabetes. It occurs as the body tries to eliminate the excess glucose through urine. Polyuria is often accompanied by increased thirst (polydipsia) and can contribute to dehydration if fluid intake is not sufficient. If you are experiencing frequent urination, along with other symptoms like increased thirst, unexplained weight loss, fatigue, or blurred vision, it is important to consult with a healthcare professional for evaluation and proper diagnosis. They can provide appropriate management and help control blood glucose levels effectively.	

Responses were reviewed by two board-certified internal medicine physicians with five and 11 years of experience and were categorized into three classes using a similar classification used in the recent literature [8]. The three categories were defined as follows: (a) appropriate - provides accurate information that meets the standard of care, (b) inappropriate - answers that convey correct information but either incomplete or outdated, and (c) unreliable - the response provides inconsistent content that does not meet the standard of care. In case of a disagreement between the two physicians, a third board-certified internal medicine physician was consulted for consensus review.

## Results

**Figure 1:**
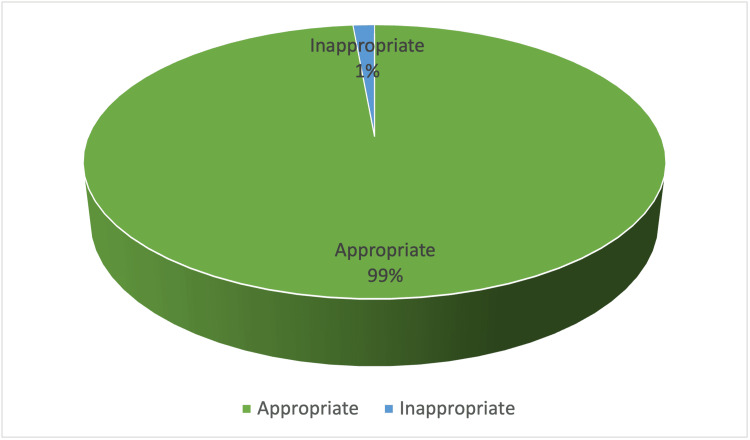
Appropriateness of answers provided by ChatGPT for type 2 diabetes education. ChatGPT: Chat Generative Pre-trained Transformer

Of the 110 questions that were initially proposed, 52.4% (40/110) were excluded after undergoing primary review regarding relevancy by physicians experienced in the field. The remaining 63.6% (70/110) of the questions were comprised of the final dataset, of which 98.5% (69/70) of the answers were categorized as appropriate, indicating that the AI model provided accurate information meeting the standard of care for type 2 diabetes management and its associated complications (Table 2). On the other hand, 1.4% (1/70) of the responses were deemed inappropriate, containing errors but still meeting the minimal standard of care in addressing the questions (Figure 1).

**Table 2:** Evaluation of ChatGPT recommendation for questions about diabetes prevention and screening based on assessment by Board-Certified Internal Medicine Physicians. ChatGPT: Chat Generative Pre-trained Transformer; COVID: coronavirus disease 2019

Question	Grade
Can type 2 diabetes be prevented?	Appropriate
Is type 2 diabetes a lifelong condition?	Appropriate
Can diabetes (DMT2) be inherited?	Appropriate
Can stress affect my blood sugar levels?	Inappropriate
Can type 2 diabetes cause changes in appetite?	Appropriate
Can type 2 diabetes cause frequent urination?	Appropriate
Can type 2 diabetes cause dry mouth?	Appropriate
Can type 2 diabetes cause headaches?	Appropriate
Can type 2 diabetes affect my vision?	Appropriate
Does type 2 diabetes increase the risk of developing severe symptoms from COVID?	Appropriate
Does type 2 diabetes increase the chance of having a heart attack?	Appropriate
Can type 2 diabetes affect my kidney?	Appropriate
Can type 2 diabetes affect my nerves?	Appropriate
Can type 2 diabetes cause intestinal problems?	Appropriate
Can type 2 diabetes cause sexual dysfunction?	Appropriate
Can type 2 diabetes cause bladder dysfunction?	Appropriate
Can type 2 diabetes increase the risk of stroke?	Appropriate
Can type 2 diabetes cause ketoacidosis?	Appropriate
How is diabetic ketoacidosis diagnosed?	Appropriate
Can type 2 diabetes affect my fertility or pregnancy?	Appropriate
Can type 2 diabetes cause vision problems?	Appropriate
Can type 2 diabetes affect my mental health?	Appropriate
Can type 2 diabetes cause foot ulcers?	Appropriate
Can type 2 diabetes cause skin problems?	Appropriate
Can type 2 diabetes cause hair loss?	Appropriate
Can type 2 diabetes cause high blood pressure?	Appropriate
Can type 2 diabetes cause urinary tract infections?	Appropriate
Can type 2 diabetes cause muscle weakness?	Appropriate
Can type 2 diabetes cause weight loss?	Appropriate
Can type 2 diabetes cause tingling or numbness in the hands and feet?	Appropriate
Can type 2 diabetes cause dizziness?	Appropriate
Can type 2 diabetes cause osteoporosis?	Appropriate
Can type 2 diabetes cause memory problems?	Appropriate
Can type 2 diabetes cause delayed wound healing?	Appropriate
Can type 2 diabetes cause dental procedure complications?	Appropriate
Should I check my blood pressure regularly if I am diagnosed with type 2 diabetes?	Appropriate
Should I have regular eye examinations if I have type 2 diabetes?	Appropriate
Is it necessary to wear closed-toe shoes with type 2 diabetes?	Appropriate
Can medications for type 2 diabetes treatment cause hypoglycemia?	Appropriate
Can I get off my diabetic medications if I lose weight?	Appropriate
Would having bariatric surgery cure my type 2 diabetes?	Appropriate
How can I lower my chances of a heart attack or stroke if I have type 2 diabetes?	Appropriate
Should I check my blood glucose level with every meal?	Appropriate
Should I go to the ER if my glucose levels exceed 500 mg/dL?	Appropriate
Can I eat fruits if I was diagnosed with type 2 diabetes?	Appropriate
Is an artificial pancreas the same as an insulin pump?	Appropriate
Can supplements cure my type 2 diabetes?	Appropriate
Can a vegan diet cure my type 2 diabetes?	Appropriate
Do I need to follow a special diet for type 2 diabetes?	Appropriate
Can I take my diabetes medication with my blood pressure medication?	Appropriate
Is it essential to exercise regularly if I have type 2 diabetes?	Appropriate
Can I manage type 2 diabetes through lifestyle changes alone?	Appropriate
Should I avoid food and drinks with sugar?	Appropriate
Can I eat fruits if I have type 2 diabetes?	Appropriate
Is it necessary to quit smoking if I have type 2 diabetes?	Appropriate
Is it necessary to quit alcohol if I have type 2 diabetes?	Appropriate
Is it essential to maintain a healthy weight with type 2 diabetes?	Appropriate
Should I avoid all carbohydrates if I have type 2 diabetes?	Appropriate
Should I see a dietitian for meal planning with type 2 diabetes?	Appropriate
Should I avoid caffeine if I have type 2 diabetes?	Appropriate
Is it necessary to wear sunscreen if I have type 2 diabetes?	Appropriate
Should I have a yearly flu shot if I have type 2 diabetes?	Appropriate
Should I receive the pneumococcal vaccine if I have type 2 diabetes?	Appropriate
How often should I see my primary care provider or endocrinologist if I have type 2 diabetes?	Appropriate
What is hemoglobin A1C test?	Appropriate
What level of hemoglobin A1C is considered prediabetic?	Appropriate
What level of hemoglobin A1C is considered to be diabetic?	Appropriate
What are the benefits of continuous glucose monitoring (CGM)?	Appropriate
Is it necessary to have regular dental check-ups if I have type 2 diabetes?	Appropriate
Is it necessary to check my cholesterol levels regularly if I have type 2 diabetes?	Appropriate

## Discussion

While primarily focusing on T2DM, this study was conducted in line with ongoing efforts to uncover the potential usefulness of the TGAI as a patient-education tool. Considering the prevalence of T2DM as a major public health concern, particularly in light of the widespread dependence of patients on online resources for health-related information, this study was both timely and relevant [8,9].

Indeed, the majority of responses (98.6%) by far were appropriate, which aligns closely with the findings of previous studies conducted by Haver et al. (88%) [5], Ayers et al. where out of the 195 questions and responses, ChatBot (ChatGPT version 3.5; San Francisco, CA: OpenAI) responses were favored by evaluators over physician responses in 78.6% of cases [10], and Momenaei et al. (84.6%), which appears to be a common trend among using TGAI as a source of medical information [11]. This appears to be a significantly more reliable tool when compared to the reliance of patients on using search engines for medical information, as prior studies conducted by Birru et al. showed a significantly lower response accuracy of 33.3% [12]. Another study that was carried out by Bremner et al. also demonstrated a diminished accuracy of 42% when online search engines were used for obtaining information by patients [13].

It's paramount to understand that while the majority of information may be accurate, the margins wherein inaccuracies exist can be potential pitfalls. Especially in the medical realm, even minimal misinformation can lead to misconceptions, which might inadvertently affect medical decisions [14]. Hence, while TGAI holds promise in the context of T2DM education, users should approach it with informed caution, ideally corroborating the information with licensed medical professionals [15].

While its quality is currently not thoroughly studied, it is possible that language models may evolve to establish a new standard for patients and healthcare professionals seeking reliable medical information in the future [16-18].

Limitations

Of the responses generated by TGAI, 1.4% were deemed inappropriate which highlights the need for continuous training and refinement of the TGAI tools, as well as vigilance in ensuring the utilization of the current medical guidelines by TGAI software when generating responses. Though negligible, some TGAI-generated responses were classified as unreliable, suggesting the need for further evaluation and improvement. Furthermore, there could be other questions that patients can ask on this topic that were not included in this study, necessitating further studies to ensure a comprehensive review of this topic.

## Conclusions

TGAI offers medical information in terms of quality and reliability. Remarkably, this study highlights the notable efficacy of TGAI in delivering reliable and standard-compliant information on T2DM. An overwhelming 98.5% of responses rendered by the model were aligned with the standard of care, a statistic that holds promise for the advancing landscape of patient education and artificial intelligence. As with previous studies, these findings could pave the way for incorporating AI-based platforms as supplementary tools for enhancing patient understanding and improving outcomes. Thus, while TGAI demonstrates potential as a supplementary tool for diabetes education, it emphasizes that while technology can assist, human expertise is still necessary. The blend of both, when used judiciously, can help in a new era of informed patient-centric care.
